# Application of dynamic expansion tree for finding large network motifs in biological networks

**DOI:** 10.7717/peerj.6917

**Published:** 2019-05-17

**Authors:** Sabyasachi Patra, Anjali Mohapatra

**Affiliations:** Department of Computer Science, International Institute of Information Technology, Bhubaneswar, Odisha, India

**Keywords:** Biological network, Network motif, Expansion tree, Subgraph, Graph isomorphism, DET

## Abstract

Network motifs play an important role in the structural analysis of biological networks. Identification of such network motifs leads to many important applications such as understanding the modularity and the large-scale structure of biological networks, classification of networks into super-families, and protein function annotation. However, identification of large network motifs is a challenging task as it involves the graph isomorphism problem. Although this problem has been studied extensively in the literature using different computational approaches, still there is a lot of scope for improvement. Motivated by the challenges involved in this field, an efficient and scalable network motif finding algorithm using a dynamic expansion tree is proposed. The novelty of the proposed algorithm is that it avoids computationally expensive graph isomorphism tests and overcomes the space limitation of the static expansion tree (SET) which makes it enable to find large motifs. In this algorithm, the embeddings corresponding to a child node of the expansion tree are obtained from the embeddings of a parent node, either by adding a vertex or by adding an edge. This process does not involve any graph isomorphism check. The time complexity of vertex addition and edge addition are *O*(*n*) and *O*(1), respectively. The growth of a dynamic expansion tree (DET) depends on the availability of patterns in the target network. Pruning of branches in the DET significantly reduces the space requirement of the SET. The proposed algorithm has been tested on a protein–protein interaction network obtained from the MINT database. The proposed algorithm is able to identify large network motifs faster than most of the existing motif finding algorithms.

## Introduction

Biological networks exhibit both global properties as well as local properties. Some of the global statistical properties are small-world property, scale-free network characteristics, power-law degree distribution, etc. Milo et al. (2002) first coined the concept of a network motif. This is treated as one of the important local property of a biological network. Network motifs are statistically over-represented patterns having significant functional properties. They constitute the basic building blocks of complex biological networks and essential for functional analysis. Detection of network motifs is a demanding task in order to define classes of networks and network homologies (Milo et al., 2002). Network motif plays a key role in understanding the modularity and the large-scale structure of a biological network (Vazquez et al., 2004). They have also been used for network superfamily classification (Milo et al., 2004a) and artificial network model for a real-world network, prediction of breast cancer survival outcome (McGee et al., 2017), cancer disease diagnosis (Gupta, Fayaz & Singh, 2016), drug repositioning (Mullen et al., 2016), analysis of functional network in diabetes patients (Li et al., 2016), etc. Network motifs act as a key feature in a wide range of applications of biological networks. Most studied functionally important network motifs are of small-size such as feed-forward-loop, Bifan, autoregulation, feedback loops, and dense overlapping regulons, etc. However, some of the large network motifs found in the protein–protein interaction (PPI) network of Human herpesvirus-8 and *Saccharomyces cerevisiae* have biological significance. One such large motif of size-10 is cited by Elhesha & Kahveci (2016), which is responsible for Kaposi’s sarcoma disease. This motif is found in the PPI network of Human herpesvirus-8. Li & Kim (2011) cited large motifs found in the PPI network of *S. cerevisiae*. A network motif consisting of 15 nodes, found in this network is responsible for transcriptional machinery and cell cycle regulation. Large network motif discovery will be helpful in the field of neuroscience. A common problem in this field is to find large clique formed by several nodes in a network of neurons (Song et al., 2005; Perin, Berger & Markram, 2011). The largest clique finding problem is an NP-hard problem (Howbert & Roberts, 2007).

Milo et al. (2004b) measure the significance of a network motif by comparing the real network to a suitably large number of randomized networks having the same degree distribution as the real network. They used a backtracking algorithm, mfinder for discovering network motifs. The exponential space complexity of this algorithm made this method incapable to deal with large motifs. Kashtan et al. (2004) improved the execution time of the motif-finding algorithm by using a sampling approach, but the results obtained are biased. Wernicke (2006) proposed a specialized algorithm ESU that could avoid redundancy in computation through proper enumeration. This method uses a third-party algorithm NAUTY (McKay, 1981) for checking isomorphism. The flexible pattern finder (FPF) algorithm (Schreiber & Schwobbermeyer, 2005) proposed three different frequency concepts for computing pattern frequency. These are F1, F2, and F3. Frequency measure F1 allows overlapping of both nodes and edges while counting the matches of a pattern. This concept does not satisfy downward closure property (Kuramochi & Karypis, 2005). This indicates that the motif frequency may increase with respect to increase in motif size. Frequency measure F2 allows edge-disjoint matches and F3 measure count completely disjoint matches of a pattern. The downward closure property is satisfied by both the frequency measures F2 and F3. In the FPF algorithm, the number of patterns grow rapidly with respect to increase pattern size. Therefore, searching all patterns systematically is a time-consuming task even for a medium-size pattern.

The algorithms discussed so far are network-centric that discover motifs for the whole network. Grochow & Kellis (2007) proposed a motif-centric algorithm, where frequency counting is done on a specific isomorphic class. This algorithm avoids unnecessary and redundant searches by mapping the query graph only on one representative of its equivalence class. The symmetry conditions are removed by adding constraints on the labeling of the vertices. Kashani et al. (2009) brought a new network-centric algorithm named Kavosh. It differs from other algorithms in that it builds an implicit tree rooted at the chosen vertex, and then generates all combinations with the desired number of nodes.

Omidi, Schreiber & Masoudi-Nejad (2009) proposed MODA, which is based on a pattern growth methodology. This is a subgraph-centric algorithm. The core idea of this algorithm is to first find the frequency of acyclic subgraphs, save the respective embeddings in memory and then use those embeddings in order to quickly find out the frequencies of cyclic subgraphs. MODA introduces the concept of the expansion tree (ET) which is static in nature and built at the beginning of the algorithm.

A novel algorithm proposed by Liang et al. (2015) named CoMoFinder to accurately and efficiently identify composite network motifs in genome-scale co-regulatory networks. CoMoFinder is developed based on a parallel subgraph enumeration strategy to efficiently and accurately identify composite motifs in large TF-miRNA co-regulatory networks.

Elhesha & Kahveci (2016) proposed a motif-centric algorithm (Elhesha–Kahveci) for finding disjoint motifs in a target network. The core idea of this method is to build a set of basic building patterns and find instances of these patterns. Then, the size of the motif increased by joining the known motifs with the instances of basic building patterns. This algorithm is able to discover large motifs up to size-15.

Lin et al. (2017) present a novel study on network motif discovery using graphical processing units (GPUs). The basic idea is to employ GPUs to parallelize a large number of subgraph matching tasks in computing subgraph frequencies from random graphs, so as to reduce the overall computation time of network motif discovery. Chen & Chen (2017) published an efficient sampling algorithm for network motif detection. However, the sampling approach may lead to a biased result.

**Figure 1:**
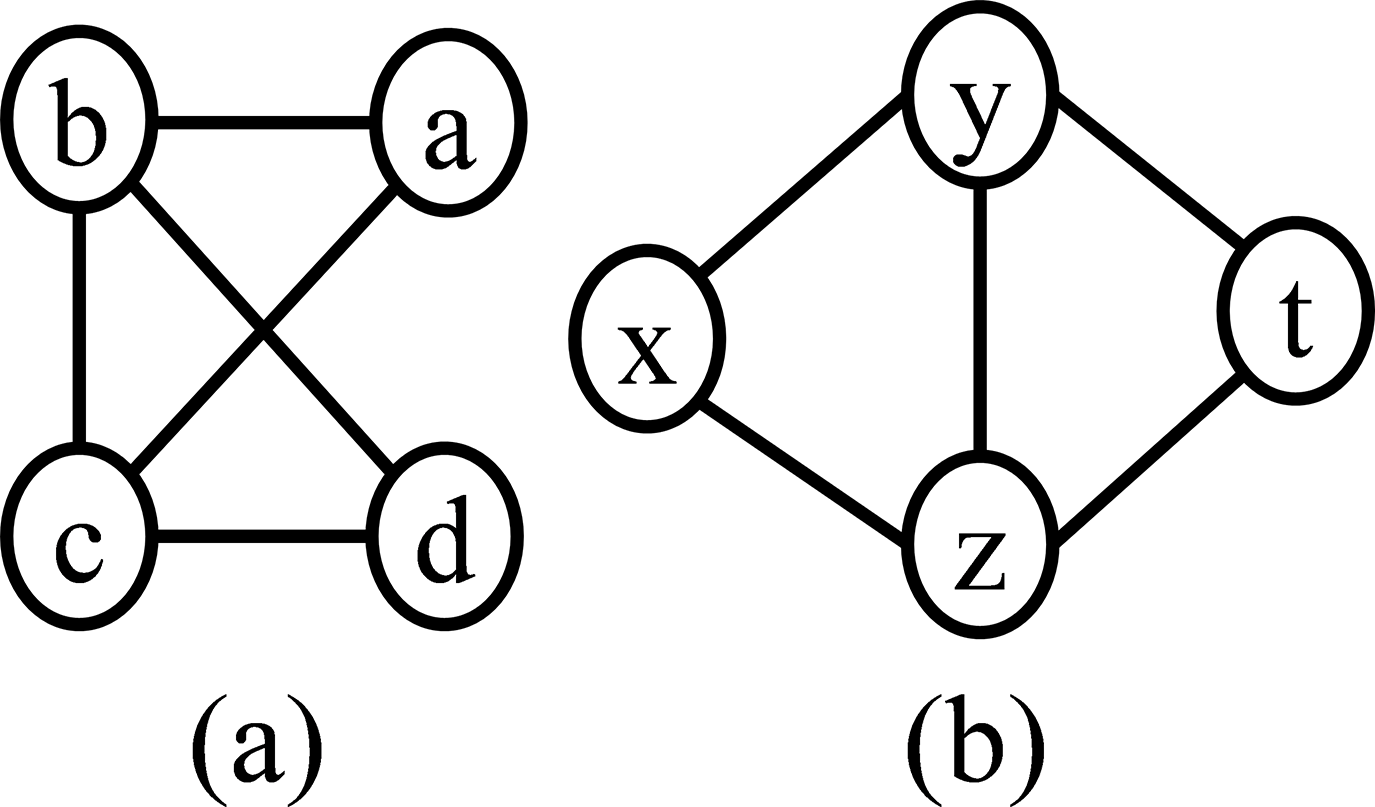
Graph (A) and graph (B) is isomorphic with each other, where the bijective function $f:V_{S_1} \to V_{S_2}$ defined as *a* → *x*, *b* → *y*, *c* → *z*, and *d* → *t*.

Network motif discovery has been proved to be a computationally hard problem (Garey & Johnson, 1990). The major challenges of this process are:

1. In order to count the frequency of a motif with known topology, it requires to solve the subgraph isomorphism problem, which is NP-Complete (Cook, 1971). Two subgraphs ${S_1} = \left({{V_S}_{_1},{E_S}_{_1}} \right)$ and ${S_2} = \left({{V_S}_{_2},{E_S}_{_2}} \right)$ of *G* are said to be identical if they have the same set of edges. A less constrained association between two subgraphs is an isomorphism. Two subgraphs *S*_1_ and *S*_2_ are isomorphic with each other under the following condition. There exists a bijection $f:{V_{{S_1}}} \to {V_{{S_2}}}$ such that $\forall \left({u,v} \right) \in {E_S}_{_1},\left({f\left(u \right),f\left(v \right)} \right) \in {E_S}_{_2}$. An example of two isomers is shown in Fig. 1. Canonical labeling is used for checking graph isomorphism. This labeling is based on the adjacency matrix representation of the graph. The adjacency matrix is ordered in a defined way so that the labeling is invariant to the initial ordering of the matrix. By comparing the canonical labeling, graphs can be checked for isomorphism. The principle of the algorithm for the generation of a canonical label is described in Kuramochi & Karypis (2005).
2. The numbers of alternative topology increase exponentially with respect to the number of edges in the motif when the motif topology is not known in advance (Tran et al., 2014; Elhesha & Kahveci, 2016).

The existing methods face major challenges when the motif size increases (Luo et al., 2018; Ciriello & Guerra, 2008; Parida, 2007; Wernicke & Rasche, 2006; Wernicke, 2005). This motivates us to design an efficient and scalable algorithm which can discover large motifs in a practical time bound. The objective of this paper is to discover large motifs present in a biological network. In order to achieve this, a motif-centric algorithm is proposed that eliminates costly isomorphic test and overcome the limitation of existing algorithms.

The central idea of the proposed method is to use a dynamic expansion tree (DET) that grows depending on the availability of the search pattern in the target network. The ET is initialized with a root node which contains a size-3 tree. Edge-disjoint subgraphs corresponding to this root node are computed first. Then the child nodes of the ET are created from the parent node by first adding vertices then edges. Vertex addition continues until the size of the subgraph reaches the desired motif size and then edges are added until a complete graph is obtained. The embeddings of a subgraph in the target network are computed along with the growth of the ET. The F2 measure is used to compute the frequency of a pattern in the target network. This frequency measure satisfies downward closure property. Hence, pruning criteria can be applied to control the growth of the ET. A branch of the ET is not expanded further when the frequency of the subgraph failed to cross a threshold. Therefore, the space requirement reduces significantly as compared to the static expansion tree (SET). The proposed method developed efficient mechanisms to avoid computationally expensive isomorphism tests during the addition of graph elements. Vertex addition can be done with time complexity *O*(*n*) and edge addition can be done approximately with time complexity *O*(1). Representation of a graph in canonical form plays a crucial role in the proposed algorithm to reduce the time complexity. Each pattern of the DET is represented in canonical form. The mapping required to convert the parent pattern to child pattern is also stored in the ET. During tree census and graph census, the embedding of a child node is directly converted to a canonical form using the stored map. This eliminates the repeated conversion of the graphs to their canonical form which is computationally very expensive. Evaluation of the proposed method using PPI networks indicates that the proposed method is significantly faster than most of the existing methods. In addition, the memory limitation of the SET is eliminated for large motif discovery.

The rest of this paper is organized as follows: The proposed method is described in the next section. Then the proposed motif finding algorithm is discussed along with computational complexity in the “Proposed Algorithm” section. Data sets, experimental results, and comparison with the existing algorithms are covered in the “Results and Discussion” section. In the “Conclusion” section, the paper is concluded with a brief conclusion and future direction.

### Motif finding using dynamic expansion tree

In this section, a motif-centric algorithm is proposed to discover large network motifs. Pattern growth approach is used in this motif-centric algorithm.

The central idea of the proposed method is to use a DET which regulates the motif finding mechanism. The root node of the ET is a minimally connected acyclic graph of three vertices (size-3 tree) and hence the number of embedding can be computed directly from the adjacency list and adjacency matrix of the target network. The ET grows in two steps; first vertices are added to the parent pattern in each successive level to reach a size-*k* tree. In this step, each node of the ET is an acyclic graph and the embeddings of these nodes are computed from the embeddings of their parent node using a tree census algorithm. In the next step, edges are added to the parent pattern in each successive level until a complete graph is obtained. In this step, the embeddings of each node are obtained by graph census algorithm. Prior to the computation of the frequency of a query graph present at a particular level of ET, the frequency of its parent must be computed and the parent embeddings are obtained from their parent and this process continues in a bottom-up manner. The frequency of a node in ET represents the number of embeddings of the subgraph in the target network. In each step, edge-disjoint embeddings are computed by using a maximum independent set (MIS) finding algorithm (Elhesha & Kahveci, 2016). A node in the ET is expanded only when the F2 frequency exceeds the predefined threshold. This pruning criterion is an implication of downward closure property of the F2 frequency measure. Hence, most of the branches of the DET vanish much before the SET. This feature of DET improves the performance of the algorithm substantially in terms of running time. In the next section, the SET, the DET and the key steps used in this algorithm are discussed.

### Static expansion tree

**Figure 2:**
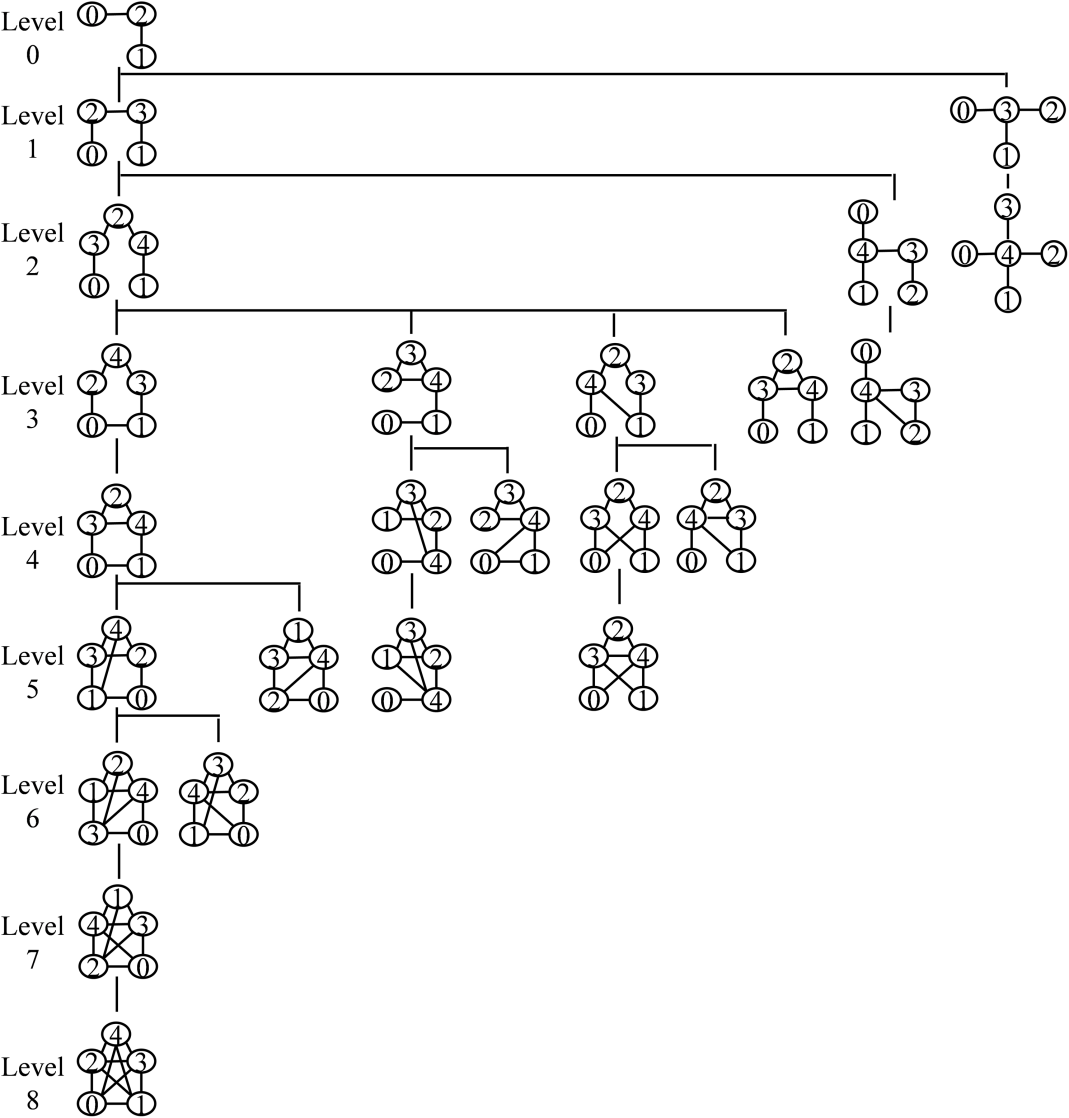
Static expansion tree *T*_5_ for size-5 motifs. The root node consists of a size-3 tree and the first level consists of two non-isomorphic size-4 trees. At the second level, there are three non-isomorphic size-5 trees and at each successive level, an edge is added to the parent graph to form a child graph. All graphs present in this tree are non-isomorphic to prevent redundancy. The depth of *T_k_* is determined by a node that holds a complete graph of *k* nodes.

The central idea of the proposed motif finding algorithm is to use an ET for searching patterns in a target network. The ET is represented by *T_k_*, where *k* represents the size of the target motif. A size-5 SET is shown in Fig. 2. A size-3 tree is present at level-0 of the ET. At the first level, there are two non-isomorphic size-4 trees, and at the second level, three non-isomorphic size-5 trees are present. Up to this level, a child graph is obtained by adding a vertex with the parent graph. Isomorphic graphs may obtain by adding a vertex with alternative parent vertices. This is elaborated in the vertex addition step. An edge is added to the parent graph to form a child graph in each successive level. Similar to vertex addition, alternative edge additions also produce isomorphic graphs. Edge addition continues until a complete graph is obtained.

### Dynamic expansion tree

**Figure 3:**
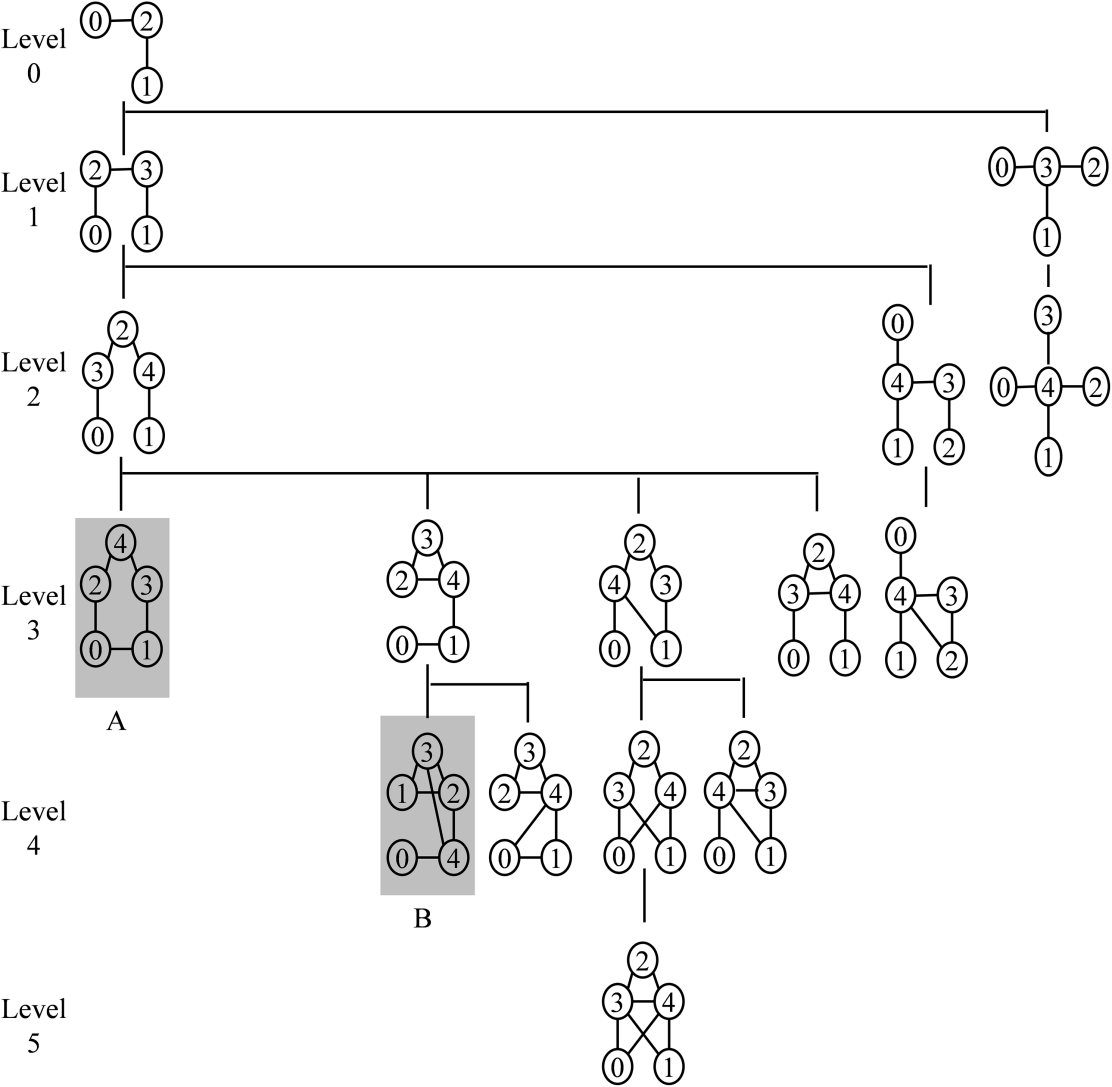
Dynamic expansion tree *T*_5_ for size-5 motifs. The root node consists of a size-3 tree and the first level consists of two non-isomorphic size-4 trees. At the second level, there are three non-isomorphic size-5 trees and at each successive level, an edge is added to the parent graph to form a child graph. All graphs present in this tree are non-isomorphic to prevent redundancy. The depth of *T_k_* is determined by threshold frequency. The shaded nodes A and B represent subgraphs whose frequency is less than the frequency threshold.

In contrast to the SET, the expansion of DET depends on the available motif instances in the target network. The DET also starts with a size-3 tree as the root node and grows similar to the SET. However, the growth is interrupted by the pruning criterion. The ET does not build a priori. A branch of this tree is expanded depending on the presence of embeddings of the pattern in the target network. In this paper, the F2 measure is used to compute the frequency of the pattern in the target network. This frequency measure satisfies the downward closure property. Hence, there is no possibility of increasing the frequency of the child pattern as compared to the parent pattern. The branch of the ET in which the node frequency is failed to cross the threshold value is pruned without further expansion. This reduces the space requirement significantly. A size-5 DET is shown in Fig. 3. The shaded nodes in the DET (Fig. 3) represent subgraphs whose appearances in the target network is less than the frequency threshold. Hence, the subtrees rooted with these nodes are pruned without further expansion.

### Vertex addition step

**Figure 4:**
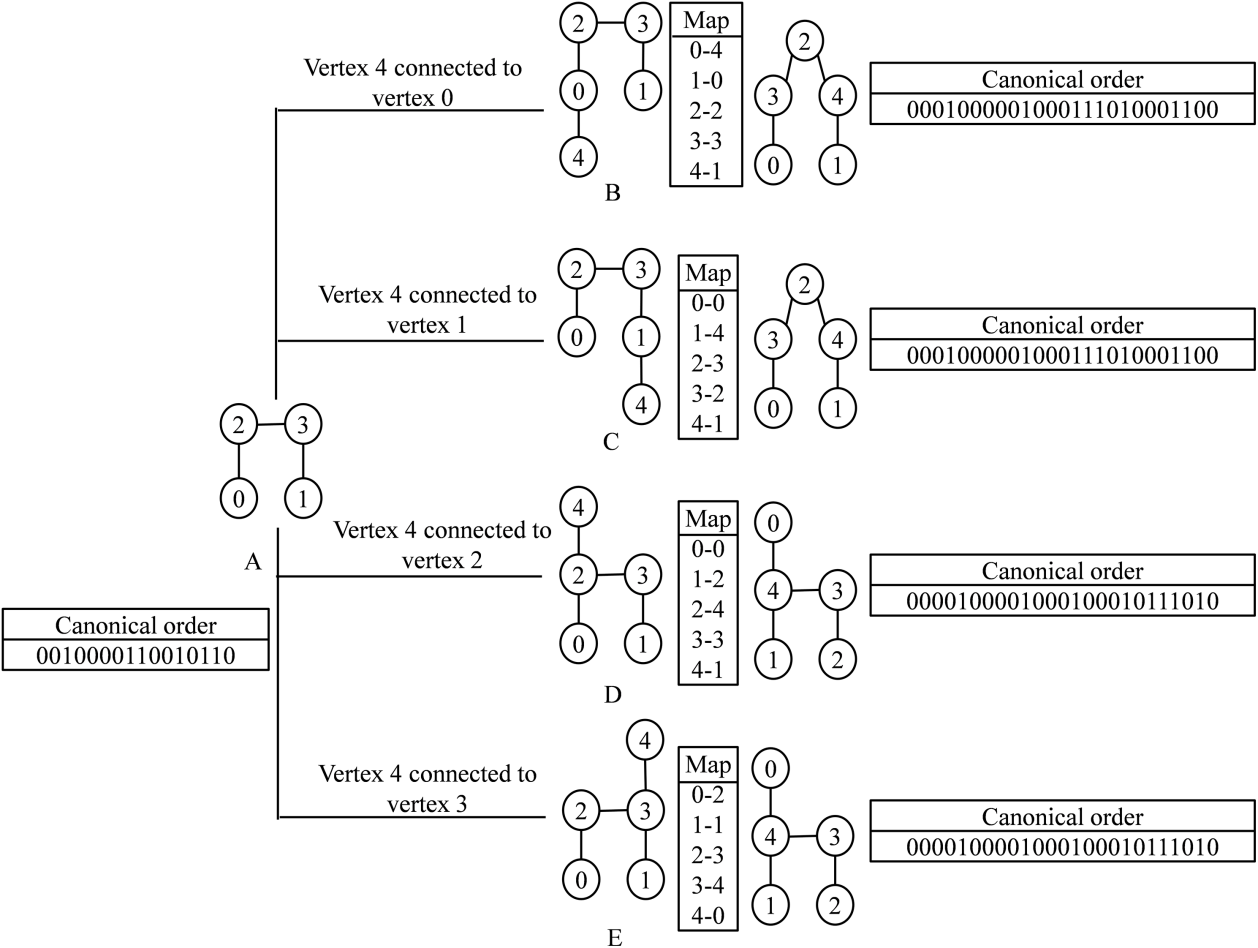
Demonstration of vertex addition step. (A) Parent graph. (B–E) Child graphs obtained after connecting a new vertex to one of the existing vertices of the parent node. Every child graphs are mapped to the canonical order. The left and the right box of each child graph represents the corresponding mapping and the canonical order.

During vertex addition, in the adjacency matrix of the parent node an extra row and an extra column are appended. Depending on the new vertex to be added, a row entry and its corresponding column are set as 1. The new tree is taken as a new child node when it is non-isomorphic to its sister nodes (from all parents). The canonical string of the child node and the mapping required to convert the resultant graph to canonical form is stored in the child node. During tree census, the embeddings of the child node are directly converted to the canonical form using the stored map. Conversion of a graph to a canonical form required only once at the time of building the ET. This reduces the time complexity significantly. In Fig. 4, tree B and tree C result after a new vertex is added to tree A. These are isomorphic with each other as the mapping leads to the same canonical order. Similarly, tree D and tree E are also isomers with each other.

### Edge addition step

**Figure 5:**
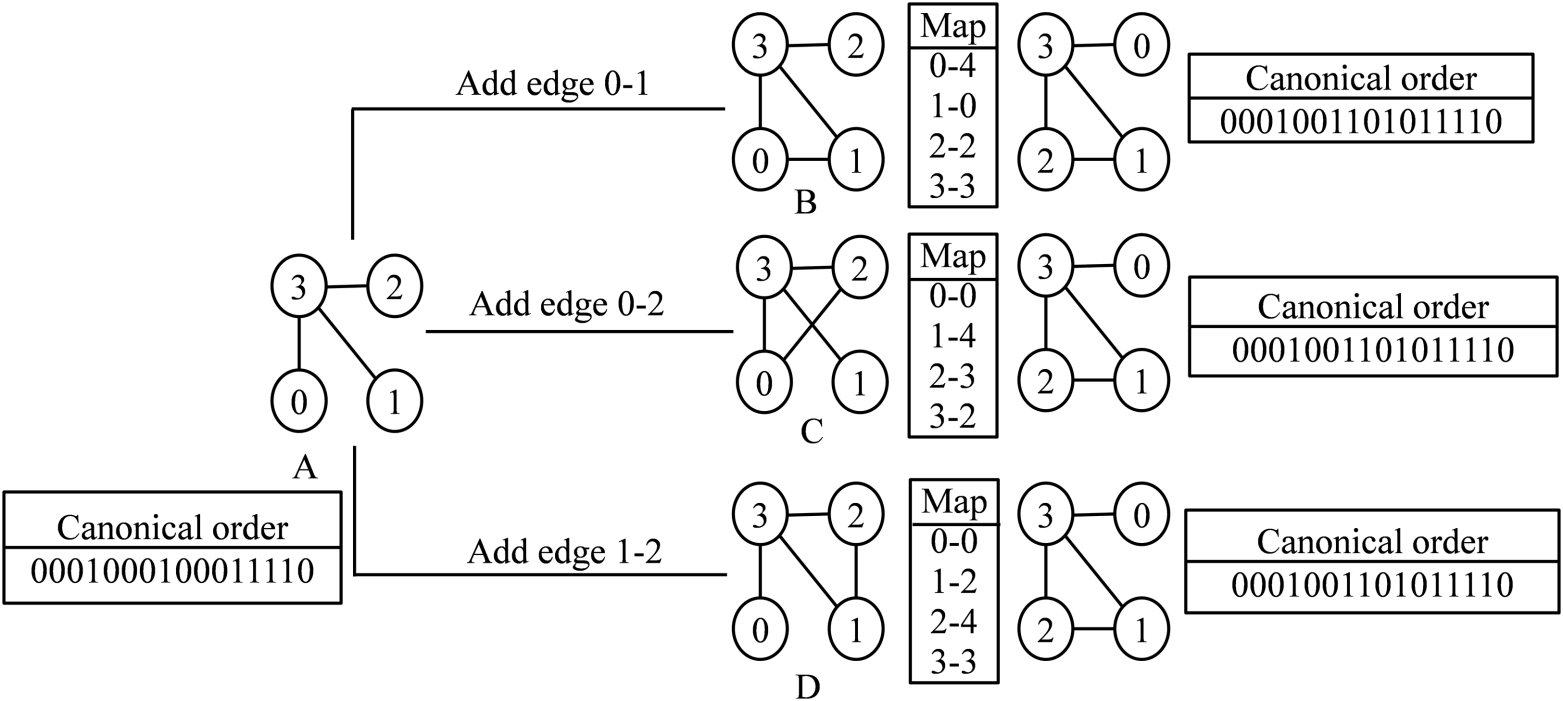
Demonstration of edge addition step. (A) Parent graph. (B–D) Child graphs obtained after edge addition. Every child graphs are mapped to the canonical order. The left and the right box of each child graph represent the corresponding mapping and the canonical order.

Edge addition can be performed by replacing a 0 by 1 in an entry of the adjacency matrix of the parent node. The new graph is taken as a new child node when it is non-isomorphic to its sister nodes (from all parents). The canonical string of the child graph and the mapping required to convert the child graph to canonical form are stored in the child node. During graph census, the embeddings of a child node are directly converted to their canonical form using the stored map. This eliminates the repeated conversion of a graph into a canonical form and the time requirement is significantly reduced. The child graphs generated by the addition of an edge with the parent graph may be isomorphic with each other as shown in Fig. 5. The isomorphic graphs are represented by a single node in the DET.

## Proposed Algorithm

**Figure 6:**
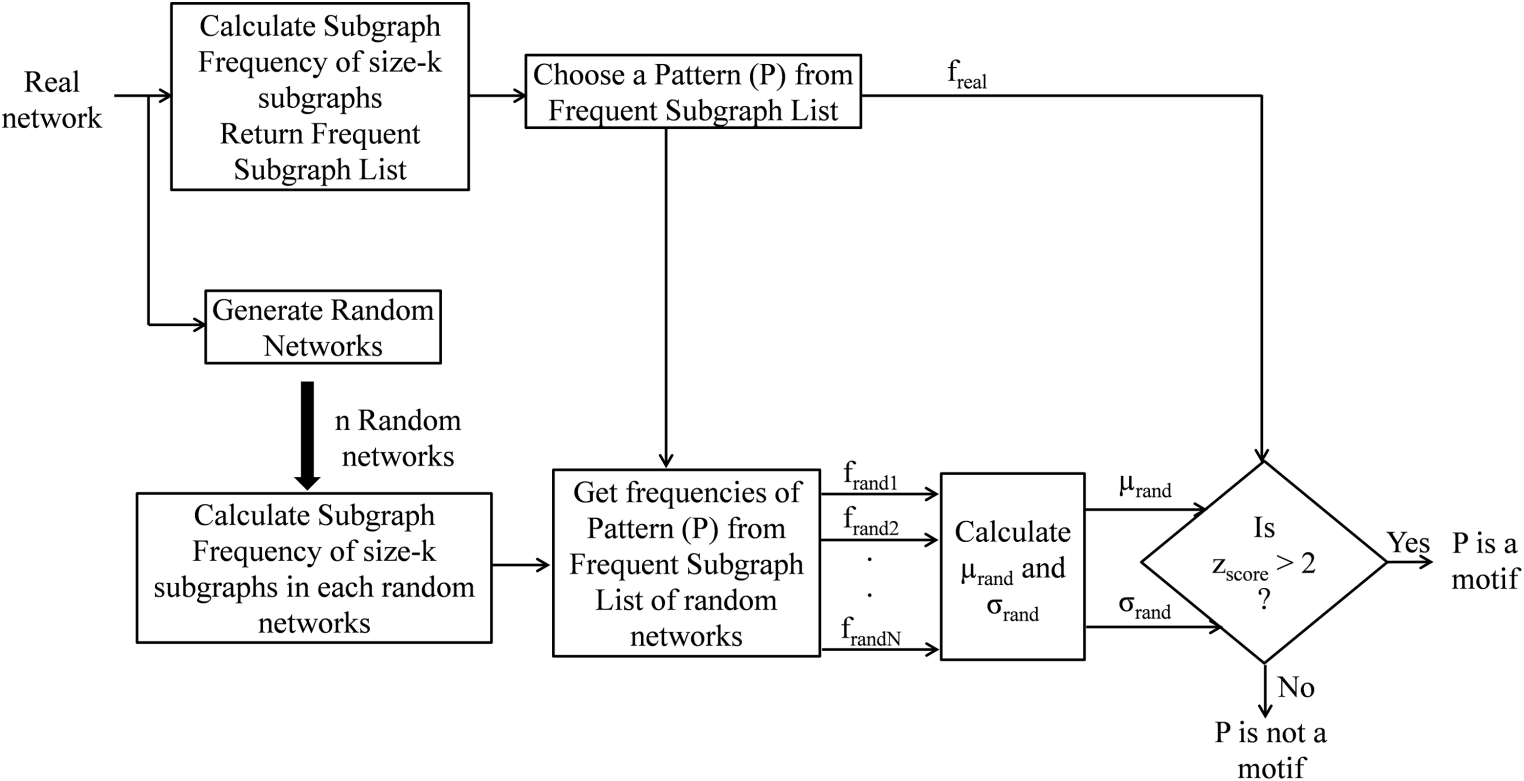
Block diagram of network motif finding method using MDET.

In this section, the proposed motif finding algorithm using a dynamic expansion tree (MDET) is explained with pseudo-code present in Algorithm 1 and a block diagram as shown in Fig. 6. MDET is used to discover statistically significant network motifs in a biological network.

**Algorithm 1:** MDET (G, N, K, Δ, F)

	**Input :** G: Target network, N: Number of randomized networks, K: Maximal network motif size, k: Subgraph size, Δ: Uniqueness threshold (*z*-score), F: Frequency threshold
	**Output :** U: Unique and frequent network motif set
**1**	**for** *k* ← 3 **to** *K* **do**
**2**	D = Calculate Subgraph Frequency (G, k, F); // D: Frequent Subgraph List in target network
**3**	**for** *i* ← 1 **to** *N* **do**
**4**	*G_rand_* = Randomized Network Generation (G); // *G_rand_*: Random network with same degree distribution as G
**5**	*D_i_* = Calculate Subgraph Frequency (*G_rand_*, k, F); // *D_i_*: Frequent Subgraph List in *i^th^* random network
**6**	**end**
**7**	**end**
**8**	*U* = Φ;
**9**	**foreach** *g* ∈ *D* **do**
**10**	s = Get Uniqueness Value (g);
**11**	**if** *s* ≥ Δ **then**
**12**	*U* =*U* ∪{*g*};
**13**	**end**
**14**	**end**
**15**	return U;

The input to the Algorithm 1 is a biological network G, a user-defined frequency threshold F, a user-defined uniqueness threshold Δ, and a user-defined maximal network motif size *K*. The output of the algorithm is a set U of repeated and unique motifs from size-3 to size *K*. The proposed algorithm consists of three major steps. First, the frequency of repeated subgraphs in the real network (line 2) is computed by using Algorithm 2. Then the frequency of the repeated subgraphs in the randomized networks (lines 3–6) is computed. Switching method is used to generate random networks (Milo et al., 2004b). Finally, the unique network motifs from the frequent subgraphs (lines 9–14) is obtained by using *z*-score. The uniqueness threshold is set as 2 and the frequency threshold is set as 5% of the size of the network. The motif size is taken up to *K* = 15 and statistical significance is measured by taking *N* = 100 random networks.

**Algorithm 2:** Calculate Subgraph Frequency (G, k, F)

	**Input** : G: Target network, k: Motif size, F: User-defined frequency threshold
	**Output** : Frequent Subgraph List: List of all subgraphs having a frequency higher than the frequency threshold
	**LocalVariable** : E: Set of embeddings of H in the target network G, *E*′: Set of edge-disjoint embeddings of H in the target network G, D: Set of size-k subgraphs present in Dynamic Expansion Tree (DET)
**1**	Initialize Frequent Subgraph List = Φ;
**2**	Construct root node of *T_k_*; // root node is a size-3 tree H
**3**	call BasicTreeEmbedding(*T_k_*, G); // Return list of embeddings E of size-3 tree H
**4**	call EdgeDisjointEmbedding(E); // Return edge disjoint embeddings *E*′ obtained from set E
**5**	**if** |*E*′| < *F* **then**
**6**	return Φ;
**7**	**end**
**8**	**if** *k* > 3 **then**
**9**	call TreeCensus(*T_k_*, G, H, *E*′, k); // return D
**10**	**else**
**11**	call GraphCensus(*T_k_*, G, H, *E*′, k); // return D
**12**	**end**
**13**	**foreach** *H* ∈ *D* **do**
**14**	**if** |*HF*| > *F* **then**
**15**	add H into Frequent Subgraph List;
**16**	**end**
**17**	**end**
**18**	return Frequent Subgraph List;

### Calculate subgraph frequency

**Figure 7:**
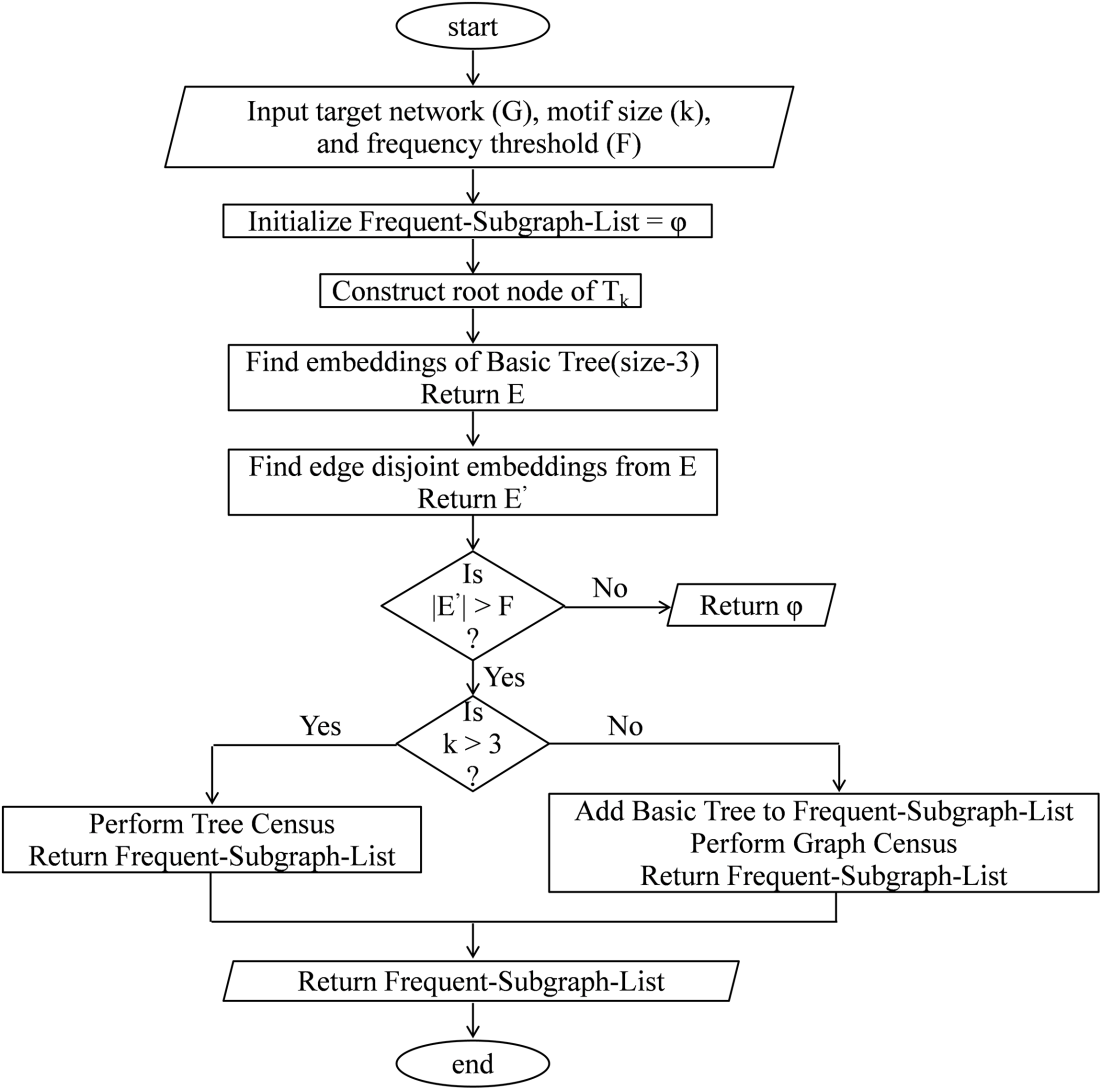
Flow chart to calculate subgraph frequency.

This module calculates the frequency of size-*k* subgraphs and returns a list of all subgraphs having a frequency higher than the frequency threshold. Along with the pseudo-code, the frequency calculation is explained with a flow chart as shown in Fig. 7. The proposed algorithm constructs the ET (*T_k_*) along with the computation of subgraph frequency. At first, the algorithm creates the root node of the DET and then fetches the size-3 query graph represented by the root node of *T_k_* and finds all its embeddings in the target network using Algorithm 3. Then, it computes the edge-disjoint embeddings by using MIS algorithm and store these calculated embeddings for future use. The DET is expanded either by adding a vertex or by adding an edge to the parent node. This expansion takes place only when the frequency of the parent node exceeds the predefined frequency threshold. After that, the query graph at the second level of *T_k_* is fetched and the frequencies of these graphs are calculated either by tree census or by graph census depending on the target motif size. Again edge-disjoint embeddings are obtained by using MIS algorithm and the pruning criterion is checked by comparing the subgraph frequency with the predefined frequency threshold. This process continues in a depth-first order till the pruning criterion is satisfied or a leaf node is obtained where there is no provision for adding new edges. The pseudo-code of the algorithm for calculating the frequency of size-*k* subgraphs is present in Algorithm 2. In this algorithm, a BasicTreeEmbedding function is called in line 3 which returns all the embeddings of the size-3 tree. Then in line 4, the EdgeDisjointEmbedding function is called which returns edge-disjoint size-3 tree list using MIS algorithm. Then, depending on the input value of *k*, either the TreeCensus function or GraphCensus function is called; lines 8–12 perform this task. If the frequency of a size-*k* subgraph is more than the user-defined frequency threshold F then that is added into the Frequent Subgraph List; lines 13–17 perform this task.

**Algorithm 3:** BasicTreeEmbedding(*T_k_*, G)

	**Input :** G: Target Network, *T_k_*: Expansion tree of size-k
	**Output :** H: size-3 tree, E: List of all embeddings of H
**1**	E = Φ;
**2**	H = root(*T_k_*);
**3**	**foreach** *u* ∈ *G.V* **do**
**4**	**foreach** *v* ∈ *Neighbour*(*u*)*, w* ∈ *Neighbour*(*u*) *and v* < *w* **do**
**5**	*e* =< *v*,*w*,*u* >;
**6**	add e into E;
**7**	**end**
**8**	**end**
**9**	return H and E;

### Basic tree embedding

In this function, all the subgraphs isomorphic to the root node of *T_k_* are obtained. In Algorithm 3, the vertex set of the graph G is denoted as G.V and the neighboring vertices of a vertex u in the underlying network denoted as Neighbour(u). In line 1 the set of all embeddings of the basic tree is initialized to empty set. All the subgraphs of the underlying network are added to set E; lines 3–8 perform this task.

### Tree census

**Figure 8:**
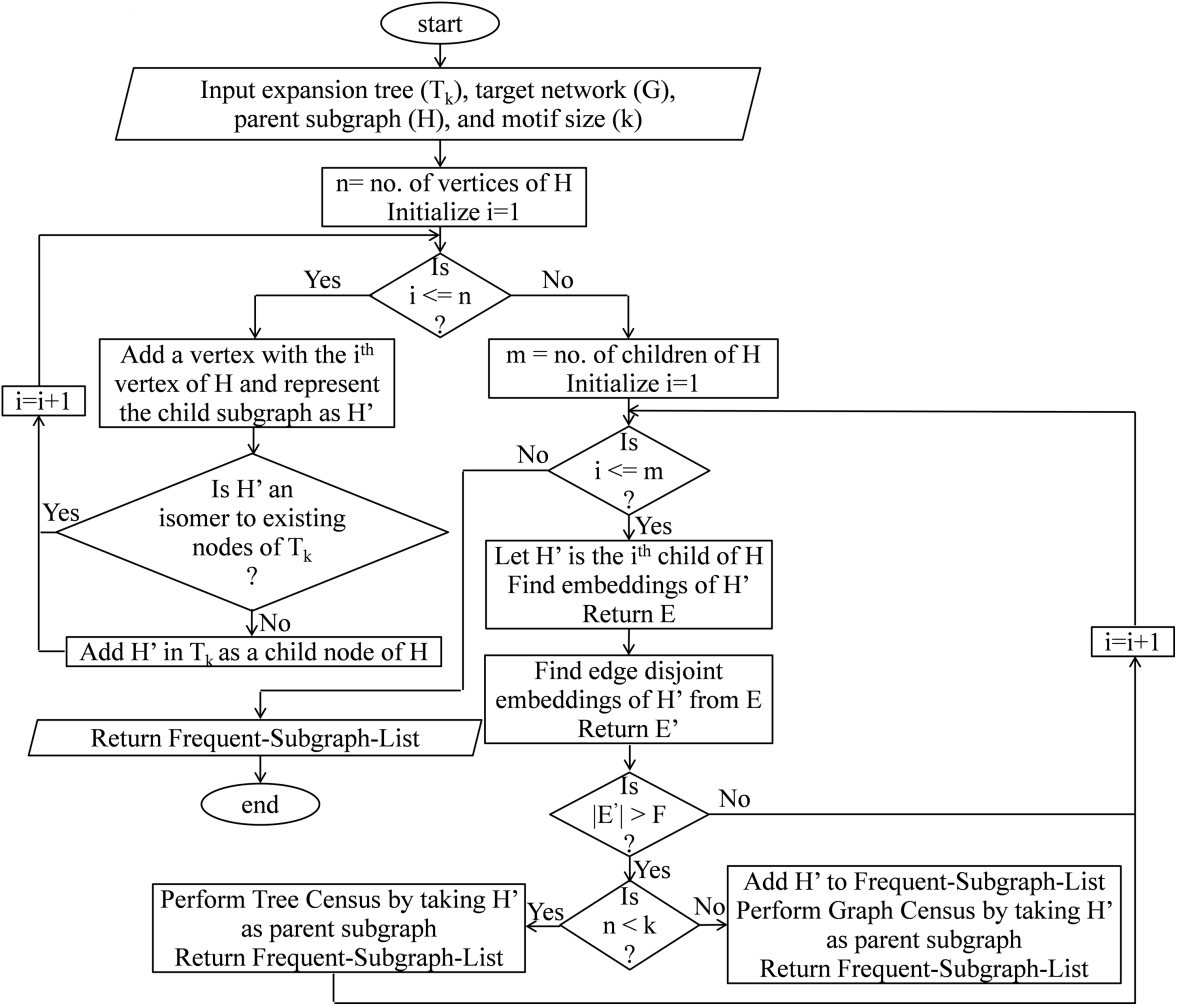
Flow chart of tree census.

This module finds a list of all subgraphs isomorphic to the child node using the embeddings of parent node where the child node has an extra vertex and an extra edge than the parent node. This procedure can be divided into two phases: (1) construction phase; and (2) expansion phase. In the construction phase, non-isomorphic children are generated from the parent node using vertex addition. In the expansion phase, the frequency of each child is computed and called for expansion if the frequency exceeds the threshold. Suppose we want to calculate the frequency of a query graph *H*′, we can extract all the embeddings represented by set E corresponding to its parent node H, then enumerate all embeddings in E that can support G and *H*′ then store them in *E*′. Let (*u*, *v*) be a new edge in *H*′ and there exists a vertex f(v) adjacent to f(u) in the target network G, then e can be added to the set *E*′. Where ${\rm{f }}:{H^\prime} \to G$. The pseudo-code of tree census is present in Algorithm 4 and the flow chart as shown in Fig. 8.

**Algorithm 4:** TreeCensus (*T_k_*, G, H, E, k)

	**Input :** *T_k_*: Expansion tree, G: Target Network, H: Parent subgraph, E: List of embedding of parent node H, k: Motif size
	**Output :** *H*′: Child subgraph, *E*′: List of embeddings of child subgraph *H*′, *E*′′: List of edge-disjoint embeddings of child subgraph *H*′
	/* Construction phase */
**1**	A = Adjacency Matrix(H);
**2**	A = Append ZeroRow and ZeroColumn(A);
**3**	n = Size(A);
**4**	**for** *i* ← 1 **to** *n* **do**
**5**	*A*′ = *A*; // Adjacency matrix of child node *H*′
**6**	*A*′(*i*,*n*) = 1 and *A*′(*n*, *i*) = 1;
**7**	**foreach** *L* ∈ *child*(*H*, *T_k_*) **do**
**8**	**if** *CheckIsomer(H*′*, L) == true* **then**
**9**	Save Map of *H*′ to L and edge difference between H and *H*′ in node L;
**10**	continue with next counter i;
**11**	**end**
**12**	**end**
**13**	if *CheckSisterIsomer(H*′, *T_k_) == true* **then**
**14**	continue with next counter i;
**15**	**end**
**16**	Create new child *H*′ in *T_k_*;
**17**	Map *A*′ to Canonical(*H*′) and save Map, Canonical order and edge difference between H and *H*′ in new node *H*′;
**18**	**end**
	/* Expansion phase */
**19**	**foreach** *H*′ ∈ *child*(*H*, *T_k_*) **do**
**20**	*E*′ = Φ;
**21**	Let (*u*, *v*) ∈ *H*′.*E* − *H.E*; // *H.E*: Set of edges of H, *H*′.*E*: Set of edges of *H*′, *u* ∈ *H*, *v* ∉ *H* and *u*, *v* ∈ *H*′
**22**	**foreach** *e* ∈ *E* **do**
**23**	Let a=f(u) where *u* ∈ *H*′, *a* ∈ *e* and *H*′ →*G*; // a is the vertex in the embedding e corresponding to u in *H*′
**24**	**foreach** *b* ∈ *Neighbour*(*a*) *and b* ∈ *G* **do**
**25**	append b in e; // if e has n vertices then *e*′ will have n+1 vertices
**26**	*e*′=Map(e); // Map is extracted from *T_k_*
**27**	add *e*′ to *E*′;
**28**	**end**
**29**	**end**
**30**	call EdgeDisjointEmbedding(*E*′); // Return edge disjoint embeddings *E*′′ obtained from set *E*′
**31**	**if** |*E*′′| < *F* **then**
**32**	continue; // Pruning subtree rooted at *H*′
**33**	**end**
**34**	**if** |*H*′.*V*| < *k* **then**
**35**	call TreeCensus(*T_k_*, G, H, *E*′′, k);
**36**	**else**
**37**	call GraphCensus(*T_k_*, G, *H*′, *E*′′, k);
**38**	**end**
**39**	**end**

Algorithm 4 returns a list of embeddings of child node H in the target network G. Child nodes of ET *T_k_* are created in lines 1–18. In line 2 an extra row and an extra column are added with the adjacency matrix of H. In line 6 a new edge is created between an old vertex and the newly added vertex. Lines 7–12 check whether the newly generated graph is an isomer to one of the children created from the same parent; if it is an isomer, then the edge difference between the parent and the new subgraph and the mapping required to convert the graph into the canonical form are saved in the existing child. Then it jumps to the next iteration. Lines 13–15 check whether the newly generated graph is an isomer to any nodes in the ET; if it is an isomer then it jumps to the next iteration. Lines 16–17 creates a new child in the ET corresponding to the new subgraph and store the canonical order of the subgraph along with edge difference between the parent and the new subgraph and the mapping required to convert the graph into the canonical form. Expansion phase starts at line 19. All the child nodes of H present in the expansion tree *T_k_* are traversed one by one. The embedding set of child subgraph *H*′ is denoted as *E*′ and it is initialized to an empty set in line 20. The extra edge needs to be added into the parent graph to obtain the child graph is denoted as (*u*, *v*). In line 22, the algorithm iterates over all the embeddings of the parent graph. Lines 23–28 generates the embeddings of the child graph from the embeddings of the parent graph. In line 30 edge-disjoint embeddings of the child node are obtained from the overlapped embeddings using MIS algorithm. If the F2 frequency of the child node failed to cross the threshold then the algorithm continues with the next child. This is shown in lines 31–33. This function recursively calls itself until the child graph size reaches to the value k otherwise graph census is called; line 34–38 perform this task.

### Graph census

**Figure 9:**
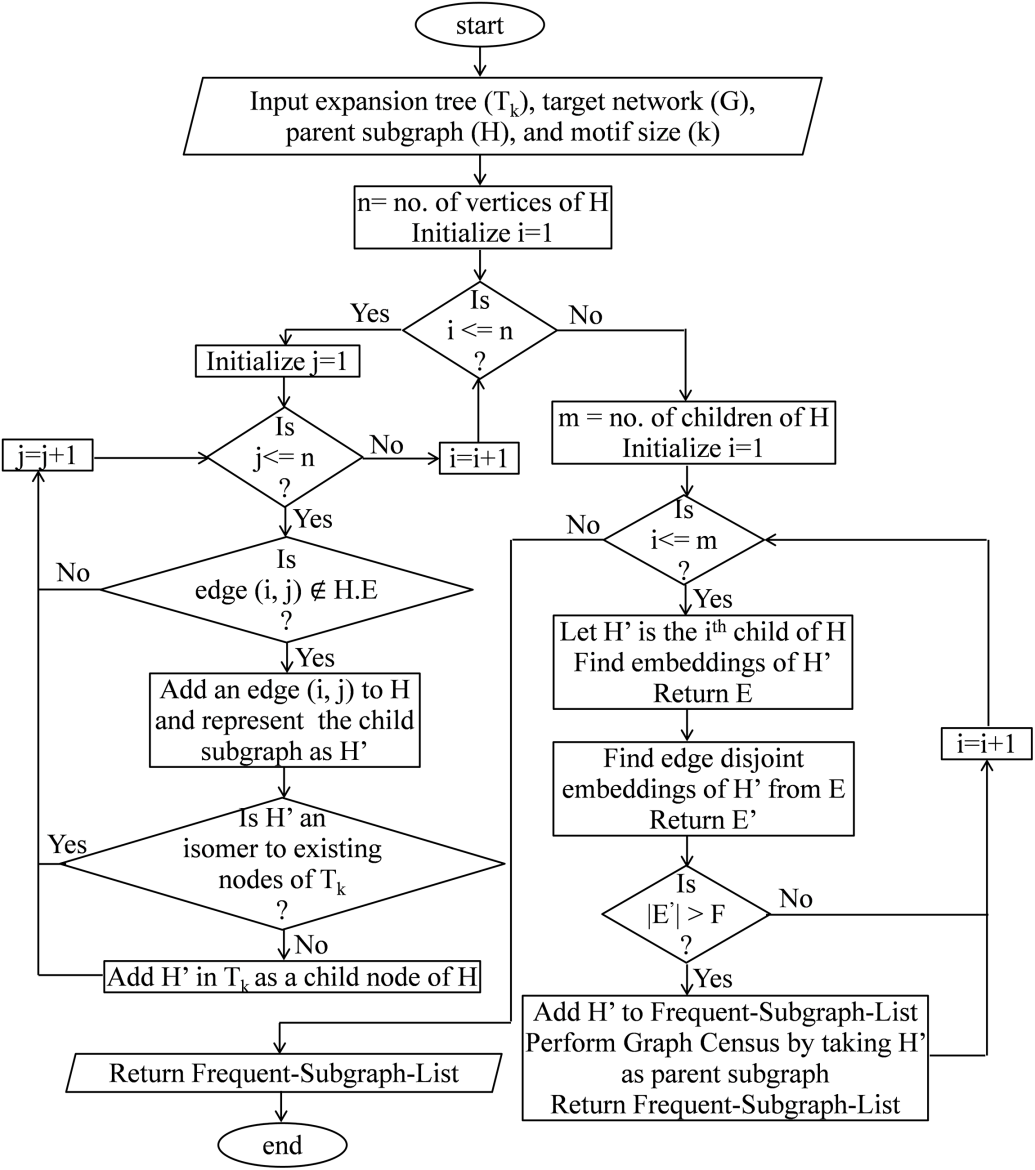
Flow chart of graph census.

This module finds a list of subgraphs isomorphic to the child node using the embeddings of the parent node, where the child node has an extra edge than the parent node. This procedure can be divided into two phases: (1) construction phase; and (2) expansion phase. In the construction phase, non-isomorphic children are generated from the parent node using edge addition. In the expansion phase, the frequency of each child node is computed and called for expansion if the frequency exceeds the threshold. Say that we want to calculate the frequency of a query graph *H*′. The embeddings (E) of parent node H extracted first, then enumerate all embeddings in E that can support G and *H*′ and store them in *E*′. Let (*u*, *v*) be a new edge in *H*′ and there exists an edge (*f*(*u*), *f*(*v*)) in the target network G, then e can be added to the set *E*′. Where $f:{H^\prime} \to G$. The pseudo-code of tree census is present in Algorithm 4 and the flow chart as shown in Fig. 9.

Algorithm 5 returns a list of embeddings of the child node H in the target network G. Child nodes of ET *T_k_* are created in lines 1–21. In line 7 a new edge is created in the adjacency matrix of child node *H*′. Lines 8–13 check whether the newly created graph is an isomer to one of the children created from the same parent; if it is an isomer, then the edge difference between the parent and the new subgraph and the mapping required to convert the graph into the canonical form are saved in the existing child. Then it jumps to the next iteration. Lines 14–16 check whether the newly generated graph is an isomer to any nodes in the ET; if it is an isomer, then it jumps to the next iteration. Lines 17–18 creates a new child in the ET corresponding to the new subgraph and store the canonical order of the subgraph along with the edge difference between the parent and the new subgraph and the mapping required to convert the graph into a canonical form. Expansion phase starts at line 22. This algorithm iterates over all the child nodes of H present in the expansion tree *T_k_*. The embedding set of child subgraph *H*′ is denoted as *E*′ and it is initialized to an empty set in line 23. The extra edge need to be added into the parent graph to obtain the child graph is denoted as (*u*, *v*); line 24 perform this task. In line 25, the algorithm iterates over all the embeddings of the parent graph. Lines 26–29 perform the task whether the addition of a new edge in the parent embedding support the target network or not. In line 27 mapping is done based on the canonical order of the resultant graph after edge addition. In line 31 edge-disjoint embeddings of the child graph are obtained from the overlapped embeddings using MIS algorithm. If the F2 frequency of the child node failed to cross the threshold then the algorithm continues with the next child. This is shown in lines 32–34. This function recursively calls itself until the child graph become a complete graph; lines 35–37 perform this task.

**Algorithm 5:** GraphCensus (*T_k_*, G, H, E, k)

	**Input :** *T_k_*: Expansion tree, G: Target network, H: Parent subgraph, E: List of embedding of parent node H, k: Motif size
	**Output :** *H*′: Child subgraph, *E*′: List of embeddings of child subgraph *H*′, *E*′′: List of edge disjoint embeddings of child subgraph *H*′
	/* Construction phase */
**1**	A = Adjacency Matrix(H);
**2**	n = Size(A);
**3**	**for** *i*←2 **to** *n* **do**
**4**	**for** *j*←1 **to** *i*−1 **do**
**5**	**if** *A(i,j) == 0* **then**
**6**	*A*′ = *A*; // Adjacency matrix of child node *H*′
**7**	*A*′(*i*, *j*) = 1 and *A*′(*j*, *i*) = 1;
**8**	**foreach** *L* ∈ *child*(*H*, *Tk*) **do**
**9**	**if** *CheckIsomer(H*′*, L) == true* **then**
**10**	Save Map of *H*′ to L and edge difference between H and *H*′in node L;
**11**	continue with next counter j;
**12**	**end**
**13**	**end**
**14**	**if** *CheckSisterIsomer(H*′, *Tk) == true* **then**
**15**	continue with next counter j;
**16**	**end**
**17**	Create new child *H*′ in *Tk*;
**18**	Map *A*′ to Canonical(*H*′) and save Map, Canonical order and edge difference between H and *H*′ in new node *H*′;
**19**	**end**
**20**	**end**
**21**	**end**
	/* Expansion phase */
**22**	**foreach** *H*′ ∈ *child*(*H*, *Tk*) **do**
**23**	*E*′ = Φ;
**24**	Let (*u*, *v*) ∈ *H*′.*E* −*H.E*; // *H.E*: Set of edges of H, *H*′.*E*: Set of edges of *H*′, *u*, *v* ∈ *H* and *u*, *v* ∈ *H*′
**25**	**foreach** *e* ∈ *E* **do**
**26**	**if** (*f* (*u*), *f* (*v*)) ∈ *G where f* : *H*′ →*G* **then**
**27**	*e*′=Map(e); // Map is extracted from *Tk*
**28**	add *e*′ to *E*′;
**29**	**end**
**30**	**end**
**31**	call EdgeDisjointEmbedding(*E*′); // Return edge disjoint embeddings *E*′′ obtained from set *E*′
**32**	**if** |*E*′′| < *F* **then**
**33**	continue; // Pruning subtree rooted at *H*′
**34**	**end**
**35**	**if** $\left| {H^\prime .V} \right|\, < {{k(k-1)} \over 2}$ **then**
**36**	call GraphCensus(*Tk*, G, *H*′, *E*′′, k);
**37**	**end**
**38**	**end**

### Computational complexity

In this section, the time complexity of the proposed method is analyzed. The complexity of the algorithms is expressed with respect to two parameters: (1) the number of vertices of the target network (*n*) and (2) motif size (*k*).

#### Algorithm 3 (Basic tree embedding)

In this step, the embeddings of the size-3 tree are generated directly from the adjacency matrix. Let *d*(*v_i_*) represents the degree of node *v_i_*. The time complexity of collecting the subgraphs isomorphic to the size-3 tree is $\sum\nolimits_{{v_i} \in V} \left({{{d({v_i})} \over 2}} \right)$. In the worst case *d*(*v_i_*) = *O*(*n*), hence the complexity can be derived as

(1) $$\sum\limits_{i = 1}^n \left({{n \over 2}} \right) = n\cdot\left({{n \over 2}} \right) = n\cdot{{n(n-1)} \over 2} = O({n^3})$$

#### Algorithm 4 (Tree census)

In the construction phase, the graph isomorphism check is done which has exponential time complexity. However, checking isomorphism is required only for creating the child nodes. This is limited in number, and once the child nodes are created no further isomorphism check required in the expansion phase. In the expansion phase, candidate vertices of the parent graph are checked for extension one by one. Let *m* is the number of candidate vertices for a possible extension where *m* lying between 1 and *k*. In order to add a vertex to a candidate vertex, all neighbors of the candidate vertex are checked one by one. Thus, the complexity of vertex addition is $\sum\nolimits_{{v_i} \in M} d({v_i})$. In the worst case *d*(*v_i_*) = *O*(*n*) and the complexity becomes *O*(*nk*) which can be approximately taken as *O*(*n*), when *k* << *n*.

#### Algorithm 5 (Graph census)

Similar to the TreeCensus here also isomorphism check is required only in the construction phase. Hence, it is also limited in numbers and does not require in the expansion phase. In the expansion phase, an edge is added to the parent graph to obtain the child graph. Let *m* is the number of candidate edges which is lying between 1 to (*k* − 1)(*k* − 2)/2. An edge can be added in *O*(1) time complexity. Thus, the complexity of edge addition is $\sum\nolimits_{{e_i} \in M} 1$. In the worst case scenario, the complexity of this algorithm becomes *O*(*k*^2^) which can be approximately taken as *O*(1).

#### Algorithm 2 (Calculate subgraph frequency)

Algorithm 3 is called only once. The TreeCensus function is called at max (*k* − 2) times for each embedding of the basic tree, but most of the embeddings do not appear in the child nodes with the increasing depth of the ET. Similarly, the GraphCensus function is called at max (*k* − 1)(*k* − 2)/2 times for each embedding of the size-*k* tree, but most of them disappear much before leaf position. In addition to that, the pruning criteria interrupt the growth of most of the branches of the ET.

## Results and Discussion

Performance of the proposed motif finding algorithm is evaluated on real networks taken from the MINT database (Chatr-Aryamontri et al., 2007). The running time and the number of motifs discovered by the proposed algorithm are evaluated across six real networks. The statistical significance of potential motifs is evaluated using *p*-value and *z*-score (Wong et al., 2011). The *z*-score is defined as $z = {{{f_{{\rm{real}}}}-{{\overline f }_{{\rm{random}}}}} \mathord{\left/ {\vphantom {{{f_{{\rm{real}}}}-{{\overline f }_{{\rm{random}}}}} {{\sigma _{{\rm{random}}}}}}} \right.} {{\rm\sigma _{{\rm{random}}}}}}$; where *f*_real_ and ${\overline f _{{\rm{random}}}}$ are the frequencies of a motif in the target network and the mean frequency of the motif in randomized networks, respectively. σ_random_ represents the standard deviation of the frequencies in the randomized networks. Higher *z*-score represents significant motif. The *p*-value represents the probability that the number of times a motif appears in a randomized network, greater than or equal to the number of times the motif appears in the target network. The lower *p*-value means significant motif. In this paper, the *F*2 measure is used to compute the motif frequency and the statistical significance of a network motif is measured using *z*-score. Performance of the proposed algorithm is compared against FANMOD, MODA, and Elhesha–Kahveci.

### Data set and implementation environment

The PPI networks of six different organisms from the MINT database are used for evaluation. The details of these networks are given in Table 1. The proposed algorithm is implemented in C++. The experiment is conducted on a machine with Intel(R), Xeon(R), E5-2670 Processor, 2.3 GHz CPU, 64 GBs of main memory, and running Redhat Linux (Version: 3.10.0) operating system. The program is run with GNU GCC compiler version 4.8.3 and the compilation flag sets are Wno-write-strings, O3, and g. The program is able to handle a maximum motif size-15 in a practical time bound.

**Table 1:** PPI networks of six different species taken from the MINT database.

Network name	Network code	Number of proteins	Number of interactions
Human herpesvirus-8	Hhv8	92	170
Human herpesvirus-1	Hhv1	176	353
*Escherichia coli*	Eco	402	727
*Helicobacter pylori*	Hpy	738	1,643
*Rattus norvegicus*	Rno	1,825	3,471
*Saccharomyces cerevisiae*	Sce	3,187	9,171

### Runtime evaluation

**Figure 10:**
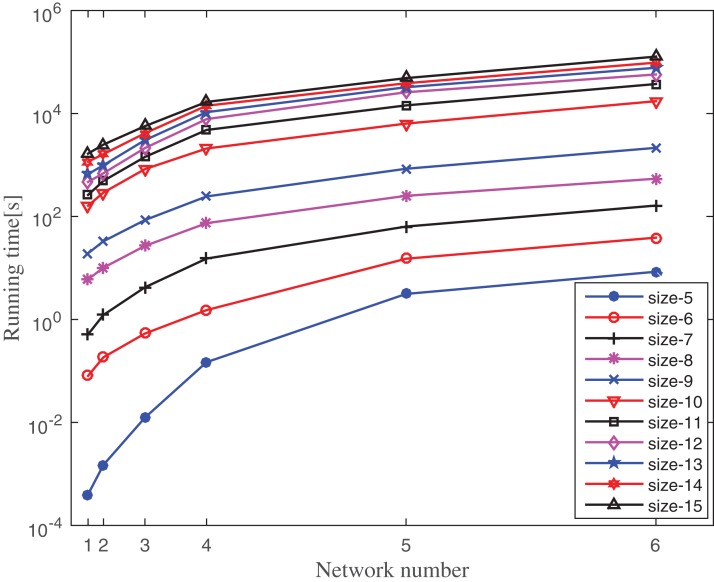
Running time of MDET for six different PPI networks by varying motif size. Network numbers from left to right along the *x*-axis represent Hhv-8, Hhv-1, *Escherichia coli*, *Helicobacter pylori*, *Rattus norvegicus*, and *Saccharomyces cerevisiae*, respectively. The position of networks along the *x*-axis depends on the network size mentioned in Table 1. The running time is measured in seconds.

In this section, the runtime of the proposed motif finding algorithm is computed on six real PPI networks. The frequency threshold is set as 5% of the size of the network and the *z*-score is set as 2. The F2 measure is used to compute motif frequency. The effect of motif size on the running time is observed by varying the motif size from 5 to 15. The experiment is repeated for 10–100 times depending on the motif size and the network size and the average running time are shown in Fig. 10. The behavior of the result is a clear indication of the scalability of the proposed algorithm with respect to graph size and motif size. The proposed algorithm takes only a few seconds to run for motif size 5–7 for all the networks selected for this experiment. For instance, the average time taken by the large network of *S. cerevisiae* is only 8.4524, 38.8153, and 163.0275 s for size-5, size-6, and size-7 motif, respectively. It takes only a few minutes to run for motif size 8 and 9 for all six networks. For instance, the average time taken by the *S. cerevisiae* network is only 9.0308 and 35.2975 m for size-8 and size-9 motif, respectively. The proposed algorithm takes only a few minutes to run for motif size 10–15 for small networks like Human herpesvirus-8, Human herpesvirus-1, and *Escherichia coli* and it is limited to a few hours for very large networks such as *Helicobacter pylori*, *Rattus norvegicus*, and *S. cerevisiae*. For instance, the average time taken by the small network of Human herpesvirus-8 is only 2.7236, 4.3665, 7.8017, 11.0834, 19.3652, and 27.7275 m for size-10, size-11, size-12, size-13, size-14, and size-15 motif, respectively, and the average time taken by the large network of *S. cerevisiae* is only 4.8204, 10.3752, 15.9310, 21.4861, 27.0475, and 35.3752 h for size-10, size-11, size-12, size-13, size-14, and size-15 motif, respectively. For higher motif size, the running time is more influenced by the motif size as compared to the size of the network. This behavior is observed due to the number of alternative patterns increases exponentially with respect to motif size. Irrespective of this limitation, the proposed method is able to discover motif up to size-15 within a practical running time.

**Figure 11:**
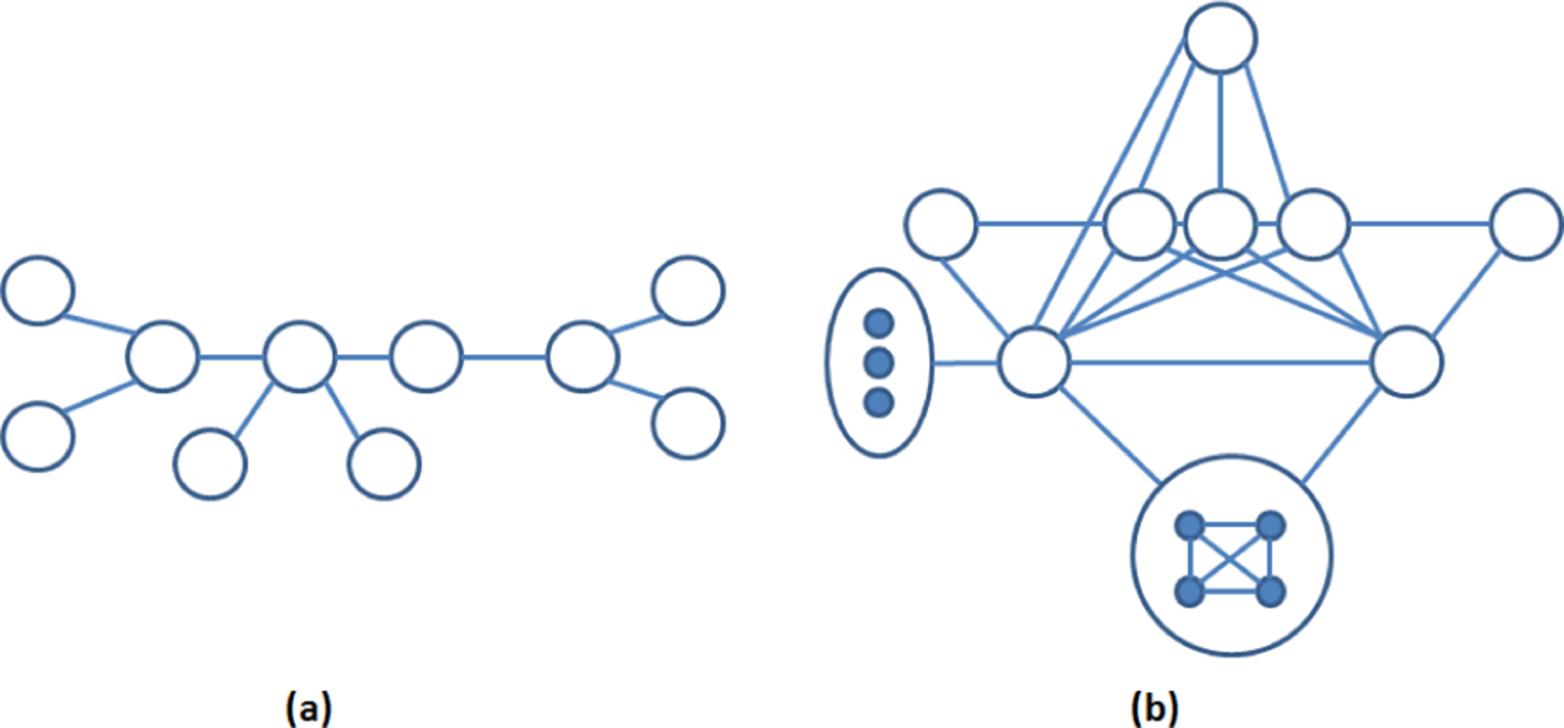
(A) A motif of 10 nodes in the left found in the PPI network of Human Herpesvirus-8 (Elhesha & Kahveci, 2016) and (B) a motif of 15 nodes in the right found in the PPI network of *S. cerevisiae*.

Table 2 contains the number of motifs found in each of the above networks by setting the frequency threshold as 5% of the size of the network. These motifs are statistically significant as they are over-represented in the target network. Some of these motifs may not be biologically significant. One of the biologically significant motifs found in the PPI network of Human herpesvirus-8 is shown in Fig. 11. This network motif of 10 nodes causes Kaposi’s sarcoma disease. Another biologically significant motif found in *S. cerevisiae* consists of 15 nodes as shown in Fig. 11. This network motif is responsible for transcriptional machinery and cell-cycle regulation in the said network.

**Table 2:** Number of significant motifs for six different PPI networks.

Motif size	Hhv8	Hhv1	*Escherichia coli*	*Helicobacter pylori*	*Rattus norvegicus*	*Saccharomyces cerevisiae*
5	10	9	11	9	8	10
10	5368	4219	5718	3241	2816	4065
15	8152	7529	8418	6719	5245	7916

### Impact of frequency threshold on MDET

In this section, the sensitivity of the proposed method with respect to the frequency threshold is discussed. The frequency threshold is computed as a percentage of order (number of nodes) of the network. A higher value of frequency threshold saturates the branches of DET much before normal saturation and a lower threshold value allow the growth of the branches even though the nodes may not represent statistically significant motif. The impact of the threshold frequency on the number of motifs found is observed in three different networks, such as Human herpesvirus-8, *E. coli*, and *S. cerevisiae*. The computed results are shown in Tables 3–5, respectively. Frequency threshold defines the number of potential motifs, which are later applied for *z*-testing to measure statistical significance. The results indicate that the number of potential motifs linearly decreases with respect to the frequency threshold. However, the number of statistically significant motifs found after *z*-test remains steady up to the frequency threshold value 5% of the size of the network. Then it decreases abruptly as some of the statistically significant motifs failed to cross the higher threshold value. Therefore, in this paper, 5% of the size of the network is taken as a standard frequency threshold value for finding network motifs.

**Table 3:** Sensitivity of MDET with respect to the frequency threshold on Human herpesvirus-8 (Hhv8) network.

Frequency threshold (% of number of nodes)	1	2	3	4	5	6	7	8	9	10
Number of potential motifs	9985	9726	9152	8671	8224	7818	7465	6640	5816	4835
Number of motifs after *z*-test	7681	7654	7628	7602	7529	7361	7102	6245	5558	4729

**Note:**

Motif size is taken as 15.

**Table 4:** Sensitivity of MDET with respect to the frequency threshold on *Escherichia coli* network.

Frequency threshold (% of number of nodes)	1	2	3	4	5	6	7	8	9	10
Number of potential motifs	9856	9526	9015	8517	7894	7182	6265	5407	4738	4135
Number of motifs after *z*-test	7018	6954	6828	6802	6719	6134	5509	5006	4496	4023

**Note:**

Motif size is taken as 15.

**Table 5:** Sensitivity of MDET with respect to the frequency threshold on *Saccharomyces cerevisiae* network.

Frequency threshold (% of number of nodes)	1	2	3	4	5	6	7	8	9	10
Number of potential motifs	10985	10426	9658	9170	8642	7915	7462	6840	6216	5315
Number of motifs after *z*-test	8124	8075	8012	7955	7916	7661	7102	6542	5968	5125

**Note:**

Motif size is taken as 15.

### Comparison with the existing methods

**Figure 12:**
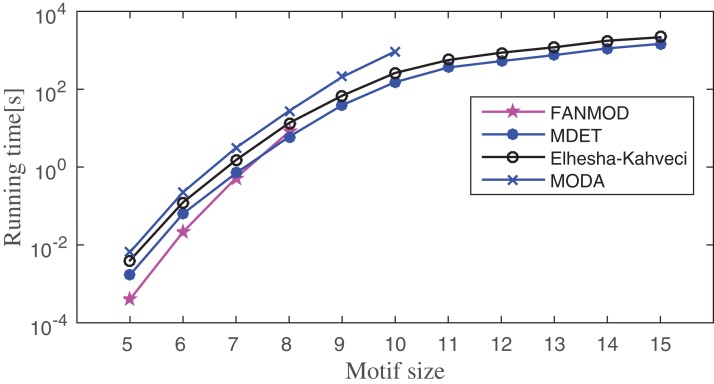
Runtime comparisons between MODA, Elhesha–Kahveci, FANMOD, and MDET in Human herpesvirus-8 (Hhv8).

**Figure 13:**
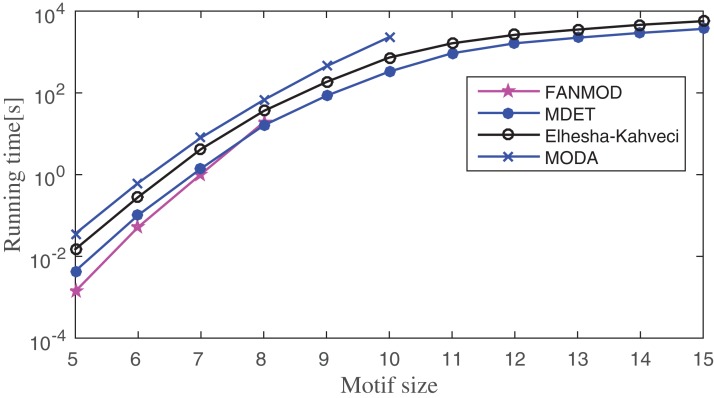
Runtime comparisons between MODA, Elhesha–Kahveci, FANMOD, and MDET in *Escherichia coli*.

**Figure 14:**
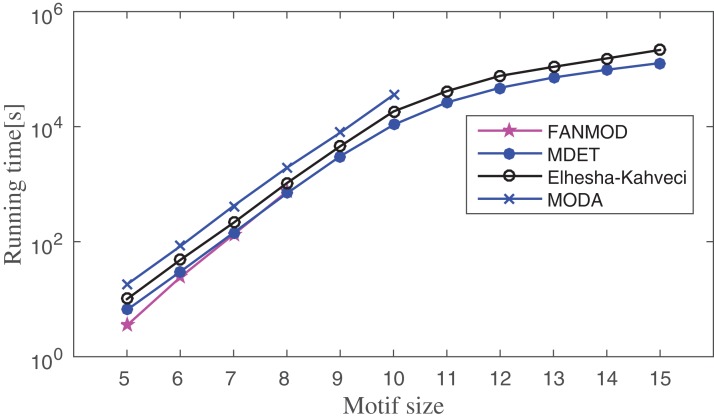
Runtime comparisons between MODA, Elhesha–Kahveci, FANMOD, and MDET in *Saccharomyces cerevisiae*.

The running time of the proposed method is compared to FANMOD, MODA, and Elhesha–Kahveci. The FANMOD and MODA count overlapping motif instances; whereas Elhesha–Kahveci and MDET count disjoint embeddings of the potential motif. In order to get disjoint embeddings of potential motifs, MIS finding algorithm is applied to the overlapping motif instances of FANMOD and MODA. The effect of this additional step on the overall running time of the above two methods is negligible. However, it makes these algorithms eligible to produce disjoint embeddings. Now all four algorithms produce disjoint embeddings and hence the comparison of the runtime is meaningful. The experiment is conducted on three PPI networks such as Human herpesvirus-8, *E. coli*, and *S. cerevisiae*. The running time is compared between these methods by varying motif size as applicable. The experiment is repeated for 10–100 times depending on motif size and network size. The average runtime is shown in Figs. 12–14 for Human herpesvirus-8, *E. coli*, and *S. cerevisiae*, respectively. These algorithms are able to determine the frequency of both induced and non-induced subgraphs.

Network motif finding problem exhibits two important characteristics; (1) the number of alternative motif topologies increases exponentially with respect to the motif size, (2) the cost of solving subgraph isomorphism also grows exponentially with respect to the size of the subgraph. Despite these two major concerns, the running time of the proposed method increases in polynomial order with respect to the motif size. Across all three networks, FANMOD and MODA are able to find motifs only up to size-8 and size-10, respectively, within a practical time bound. Elhesha–Kahveci and the proposed algorithm (MDET) are able to discover large motifs up to size-15. However, the proposed algorithm does not involve any graph isomorphism check during the census, that makes it faster as compared to Elhesha–Kahveci. A broader picture of running time ratio (RT ratio) of MDET to other algorithms are shown in Tables 6–8 for FANMOD, MODA and Elhesha–Kahveci, respectively.

**Table 6:** Running time ratio of MDET to FANMOD.

RT ratio	5	6	7	8
Hhv-8	4.25	2.95	1.32	0.74
*Escherichia coli*	3.14	1.96	1.37	0.84
*Saccharomyces cerevisiae*	1.85	1.25	1.07	0.93

**Table 7:** Running time ratio of MDET to MODA.

RT ratio	5	6	7	8	9	10
Hhv-8	0.26	0.25	0.23	0.21	0.19	0.16
*Escherichia coli*	0.23	0.20	0.18	0.17	0.15	0.12
*Saccharomyces cerevisiae*	0.38	0.34	0.31	0.27	0.23	0.18

**Table 8:** Running time ratio of MDET to Elhesha–Kahveci.

RT ratio	5	6	7	8	9	10	11	12	13	14	15
Hhv-8	0.67	0.66	0.64	0.61	0.58	0.56	0.53	0.50	0.47	0.45	0.42
*Escherichia coli*	0.61	0.58	0.54	0.51	0.49	0.46	0.44	0.41	0.36	0.33	0.29
*Saccharomyces cerevisiae*	0.66	0.67	0.65	0.63	0.62	0.60	0.59	0.57	0.56	0.54	0.52

The RT ratio between FANMOD and MDET indicates that though FANMOD performs better than MDET for motif size 5 and 6, it is closed to 1 for motif size 7 and 8. The reason for the higher run time of MDET is it takes extra time to build the ET. However, this extra time is negligible for the higher motif size. The RT ratio of MDET to MODA indicates the superiority of MDET as the ratio is in between 0.1 and 0.4 and hence MDET takes only 10–40% time of MODA depending on the motif size. The RT ratio of MDET to Elhesha–Kahveci indicates that MDET takes approximately 50% time of Elhesha–Kahveci. The RT ratio gradually decreases which indicates that the relative performance increases with increase motif size.

The MODA algorithm uses the SET and hence runs out of space long before it runs out of time. MDET uses the DET, hence this problem is abolished. This fact can be demonstrated with the help of Tables 9 and 10.

**Table 9:** Number of nodes in the static expansion tree.

Motif size (*k*)	Cumulative number of non-isomorphic trees of size 3 to *k*	Number of non-isomorphic graphs	Number of nodes in expansion tree
3	1	2	2
4	3	6	7
5	6	21	24
6	12	112	118
7	23	853	865
8	46	11117	11140
9	93	261080	261126
10	199	11716571	11716664
11	434	1006700565	1006700764
12	985	164059830476	164059830910
13	2286	50335907869219	50335907870204
14	5445	29003487462848061	29003487462850347
15	13186	31397381142761241960	31397381142761247405

**Note:**

Table is prepared using the program geng from McKay’s gtools package (McKay, 1981).

**Table 10:** Comparison between the number of nodes in the static and the dynamic expansion tree.

Motif size	3	4	5	6	7	8	9	10
Number of nodes in static expansion tree	2	7	24	118	865	11140	261126	11716664
Number of nodes in dynamic expansion tree	2	7	15	56	171	645	2158	7292

**Note:**

*Escherichia coli* network is used to prepare this table.

The total number of non-isomorphic trees starting from size-3 to size-*k* are listed in column 2. This also represents the number of internal nodes in the ET *T_k_*. The number of non-isomorphic subgraphs is listed in column 3. The total number of nodes in the ET is obtained by adding column 3 with the previous row entries of column 2. It can be observed that up to motif size-10, the space requirement of the ET is less than 1 GB. But beyond motif size-10, the space requirement increases exponentially, and it is impractical to build a static tree for running MODA. However, in a DET, the nodes are generated on-demand basis. Hence it is quite less than the number of nodes specified in Table 9.

In Table 10, a comparison between the number of nodes present in the static and the DET of the *E. coli* network is given. The number of nodes present in the DET of all six networks for the size-15 motif is given in Table 11. These numbers are quite less in compared to the SET which is 31397381142761247405 for the size-15 motif. In order to obtain these tables, the uniqueness threshold is set as 5% of the size of the network and the F2 frequency measure is used which satisfies downward closure property. Thus, a branch in the ET does not expand further if the frequency of the subgraph fails to cross the threshold value. Therefore, most of the branches of the DET pruned well before the maximal depth in contrast to the SET. It is observed from Table 9 that the number of nodes in the SET increases exponentially with respect to the motif size whereas in the case of the DET it increases linearly. Hence space limitation can be eliminated in the MDET method by the use of DET.

**Table 11:** Number of nodes in the dynamic expansion tree of various networks.

Network name	Hhv8	Hhv1	*Escherichia coli*	*Helicobacter pylori*	*Rattus norvegicus*	*Saccharomyces cerevisiae*
Number of nodes in dynamic expansion tree	12852	11297	13418	9713	8525	11791

**Note:**

Motif size is taken as 15.

## Conclusion

In this paper, the dynamic expansion tree (MDET) is used to find large motifs in the biological networks. The novelty of the proposed algorithm is that it avoids computationally expensive graph isomorphism test and overcome the space limitation of the SET. A key feature of this algorithm is that the root of the ET always started with a size-3 tree and it is expanded iteratively by addition of graph elements in each successive level. The F2 measure is used to compute the frequency of the pattern in the target network. This frequency measure satisfies downward closure property. Hence, pruning criteria can be applied to control the growth of the ET. A branch of the ET is not expanded further when the frequency of the subgraph failed to cross a predefined threshold. This reduces the space requirement significantly as compared to the SET. The representation of the graph in canonical form plays a crucial role in the proposed algorithm to reduce the time complexity. During the tree census and the graph census, the embeddings of the child node are directly converted to the canonical form using the stored map. This eliminates the repeated conversion of the graphs to their canonical form which is computationally very expensive. The pattern growth approach is used in this motif-centric algorithm that eliminates costly isomorphism tests. The running time of the proposed algorithm is evaluated by varying the motif size and the size of the target network. The implementation results on the PPI networks from MINT database indicate that the proposed algorithm is significantly faster than most of the existing motif finding algorithms. The proposed algorithm is able to discover large motifs up to size-15 within a few hours. The DET eliminates the memory limitation of the SET. But the space requirement can be further reduced by taking a balanced DET instead of a simple ET. Network motif finding using a balanced DET can be explained in the future.

## Supplemental Information

10.7717/peerj.6917/supp-1 Supplemental Information 1 This README file can be used as an user manual to run MDET. The MDT source code is available at GitHub link https://github.com/sabyasachipatra. This code accepts an input network in Simple Interaction Format (SIF). A sample network is uploaded at the GitHub link for reference.

## Additional Information and Declarations

### Data Availability

The following information was supplied regarding data availability: Data is available at GitHub: https://github.com/sabyasachipatra/modet
